# Using plant growth modeling to analyze C source–sink relations under drought: inter- and intraspecific comparison

**DOI:** 10.3389/fpls.2013.00437

**Published:** 2013-11-05

**Authors:** Benoît Pallas, Anne Clément-Vidal, Maria-Camila Rebolledo, Jean-Christophe Soulié, Delphine Luquet

**Affiliations:** ^1^Unité Mixte de Recherche Amélioration Génétique et Adaptation des Plantes Méditerranéennes et TropicalesMontpellier SupAgro, Montpellier, France; ^2^Unité Mixte de Recherche Amélioration Génétique et Adaptation des Plantes Méditerranéennes et Tropicales, Centre de Coopération Internationale en Recherche Agronomique pour le DéveloppementMontpellier, France

**Keywords:** drought, plant biomass accumulation, non-structural carbohydrate, source and sink regulation, functional structural plant model, rice, oil palm

## Abstract

The ability to assimilate C and allocate non-structural carbohydrates (NSCs) to the most appropriate organs is crucial to maximize plant ecological or agronomic performance. Such C source and sink activities are differentially affected by environmental constraints. Under drought, plant growth is generally more sink than source limited as organ expansion or appearance rate is earlier and stronger affected than C assimilation. This favors plant survival and recovery but not always agronomic performance as NSC are stored rather than used for growth due to a modified metabolism in source and sink leaves. Such interactions between plant C and water balance are complex and plant modeling can help analyzing their impact on plant phenotype. This paper addresses the impact of trade-offs between C sink and source activities and plant production under drought, combining experimental and modeling approaches. Two contrasted monocotyledonous species (rice, oil palm) were studied. Experimentally, the sink limitation of plant growth under moderate drought was confirmed as well as the modifications in NSC metabolism in source and sink organs. Under severe stress, when C source became limiting, plant NSC concentration decreased. Two plant models dedicated to oil palm and rice morphogenesis were used to perform a sensitivity analysis and further explore how to optimize C sink and source drought sensitivity to maximize plant growth. Modeling results highlighted that optimal drought sensitivity depends both on drought type and species and that modeling is a great opportunity to analyze such complex processes. Further modeling needs and more generally the challenge of using models to support complex trait breeding are discussed.

## INTRODUCTION

Under drought as under other abiotic constraints, plants adjust their functioning to optimize the access to the limiting resource. Such adjustments were extensively studied and gave rise to the “functional equilibrium” theory about 30 years ago (Brouwer, 1983). Thereafter it was associated to the notion of plant phenotypic plasticity (Nicotra and Davidson, 2010) that, for example, enables the plant to privilege root to the detriment of shoot growth under any soil resource deficiency: phosphorus (Shane et al., 2003; Luquet et al., 2005), water (Chaves et al., 2002), or nitrogen (Grechi et al., 2007). Such behavior involves the regulation of both C source (assimilation) and sink (organ appearance and growth rates) activities. It was reported, during a gradual soil dry-down for annual (maize; Tardieu et al., 1999) or perennial plants (grapevine; Lebon et al., 2006) that the whole plant expansive growth under moderate water deficit is down-regulated while C assimilation remains unaffected. This lack of relationship between C availability and expansive growth could be related to a reduced water flux to growing cells or to modified cell-wall mechanical proprieties driven by hormone signaling (Muller et al., 2011). However, many studies reported that already under moderate drought, non-structural carbohydrate (NSC: hexose, sucrose, starch) metabolism was modified in both source and sink organs despite the whole plant NSC balance was not affected (Luquet et al., 2008; Pantin et al., 2011). Meanwhile, several studies revealed a decrease in NSC under long and severe water deficit (McDowell et al., 2008) or when photosynthesis activity falls down close to 0, also suggesting source limitation of growth under drought.

In the last decade a series of studies were initiated on the role of NSC source–sink relationships in plant phenotypic plasticity and the agronomic performance of annual and perennial monocots under abiotic constraints, particularly drought (e.g., Dingkuhn et al., 2007). It was reported for rice that growth maintenance under drought was positively correlated with starch mobilization and that the latter was greater for genotypes with low potential growth rate and high starch storage under non-limiting conditions (Rebolledo et al., 2012, 2013). On perennial monocots as oil palm, the large pool of NSC reserves in the trunk was reported to play a major role for buffering source–sink imbalances resulting from the long period between the determination of bunch yield components (4 years before harvest) and the bunch filling period (Legros et al., 2009a; Pallas et al., 2013a). Under drought, it was observed that a decrease in palm tree development rate reduced plant assimilate demand and as a consequence carbohydrate reserve mobilization; this might be considered as poorly efficient for agronomic performance but favorable for tree survival (Legros et al., 2009c).

Such results highlight the complex relations between plant C and water balances under drought that underlie its performance (Watkinson et al., 2008; Sulpice et al., 2009). The analysis of such complexity can be supported by plant and crop modeling (Chenu et al., 2009; Tardieu and Tuberosa, 2010; Luquet et al., 2012a; Quilot-Turion et al., 2012). Several modeling approaches were proposed to take into account the multiple effects of water deficit on plant growth and C metabolism. Tardieu et al. (2011) described two approaches commonly used in plant growth models: (i) the first one considers that plant growth under water deficit is driven directly by integrative plant variables such as plant carbon status (Yan et al., 2004), (ii) the second one considers the existence of parallel mechanisms affecting plant expansive growth (hormonal and hydraulic signals) or biomass accumulation (stomatal conductance, photosynthesis) without any coordination at the plant scale. For this second approach, the modeling exercise consists in identifying relevant sub-models for each meta-process without taking into account whole plant status and related local regulations. This approach was carried out in many crop models (Brisson et al., 2003; Hammer et al., 2010). But in our opinion, this approach is not totally relevant since it does not take into account other experimental evidences showing feedbacks among plant C and water status, development, and expansive growth (Luquet et al., 2008 on rice; Pallas et al., 2013a on oil palm).

In order to assess the relative impact of C source vs. sink growth limitation on plant performance under drought, original functional structural plant models were developed. These models are dedicated to annual (Ecomeristem; Luquet et al., 2006) and perennial (X-Palm; Pallas et al., 2013b) monocots. Basically, these models rely on a detailed and dynamic representation of plant topology and morphogenesis at organ level. Plant morphogenesis is controlled by genotypic potential parameters (e.g., organ potential size and appearance rate), based on model calibration using data acquired under non-limiting conditions. It can be then down-regulated by intermediate plant variables, affecting both sink (organ size, number, C storage) and source (leaf transpiration, C assimilation) activities.

The present paper aims to explore the way the regulation of C sink and source under drought can be optimized to improve phenotype performance, i.e., biomass production. For this purpose, original experimental results or the re-interpretation of previous ones (Luquet et al., 2008; Rebolledo et al., 2012) are used. This experimental approach is combined with modeling approaches provided by Ecomeristem (rice; Luquet et al., 2006) or X-Palm (oil palm; Pallas et al., 2013b). Once presented models’ concepts, a set of simulation experiments for different virtual genotypes (sink or source limited) are presented considering different drought types of particular concern for each species: soil dry-down as observed in rainfed or lowland conditions for rice (Courtois et al., 2000) and in West African plantation for oil palm (Combres et al., 2013). Simulation results are then discussed regarding the role plant modeling can play in: (i) the analysis of C source–sink relations and their optimization with respect to a given constraint, (ii) the exploration of trade-offs between plant survival and agronomic performance.

## MATERIALS AND METHODS

### EXPERIMENTAL DESIGN

#### Rice

Two data sets, relying on the same experimental design, were used as a starting point of the present study. These data sets were extensively detailed in Luquet et al. (2008) and Rebolledo et al. (2012) and only the key principles will be reminded. In both experiments, rice plants were cropped in 1 l pot (enabling a rapid colonization by roots of the soil volume) in a greenhouse in Montpellier, France (43°39′N, 3°52′E). During these experiments two treatments were compared, a well-watered and a drought one. For the water stress treatment, watering was stopped at the stage of leaf 6 appeared on the main stem and the dry-down was maintained until fraction of transpirable soil water (FTSW; Sinclair and Ludlow, 1986) reached 0.2. FTSW was defined as the ratio of actual plant-available soil water (ASW) content to the total plant-available soil water (TTSW). TTSW was defined as the difference between soil water content at field capacity (SWC_fc_) and soil water content when leaf transpiration was negligible (SWC_min_; <10% of maximal leaf transpiration rate per unit leaf area; Pellegrino et al., 2004). ASW was defined as the difference between actual soil water content and SWC_min_. In the first experiment (Luquet et al., 2008), only one indica genotype IR64 was grown. Growth (shoot dry weight, leaf size), development (leaf number on the main stem, tillering) measurements, and sugar content analyses (hexoses, sucrose, and starch) were performed during and at the end of the dry-down. NSC concentration (mg per g of dry weight) was measured on the last fully expanded leaf (called afterward mature leaf) and pale green hidden expanding leaves on the main stem (called afterward young leaves). Young leaves are considered as sinks for C assimilates, and the mature leaf as a source of C assimilates for plant growth. In the second experiment (Rebolledo et al., 2012), 43 genotypes (mainly japonica types) were studied and the same growth and sugar content analyses were performed only at the final sampling (FTSW = 0.2) for both treatments.

#### Oil palm

One experiment was carried out between October 2011 and January 2012 in a greenhouse on potted oil palm (5 l filled with organic compost) in Montpellier, France. Two high-yielding tenera hybrids, called afterward G01 and G02 were studied. The plants were 6 months old at the onset of the experiment. During the experiment mean daily temperature, photosynthetic photon flux density (PPFD) and vapor pressure deficit (VPD) were respectively equal to 26.6°C, 16.8 mol m^-^^2^ d^-^^1^and 2.4 kPa.

Water treatments were applied 6 months after germination when plants displayed a number of visible leaves close to 6. At this stage the root system entirely colonized the pot volume. During the experiment, the relative transpiration (Tr_norm) of the water stressed plants was estimated as the ratio of plant transpiration per unit leaf area to the mean value of the transpiration per unit leaf area for control plants. Pots were placed in plastic bags to avoid soil evaporation. Thus, plant daily transpiration was estimated as the water loss of the pot between two consecutive days.

**FIGURE 1:**
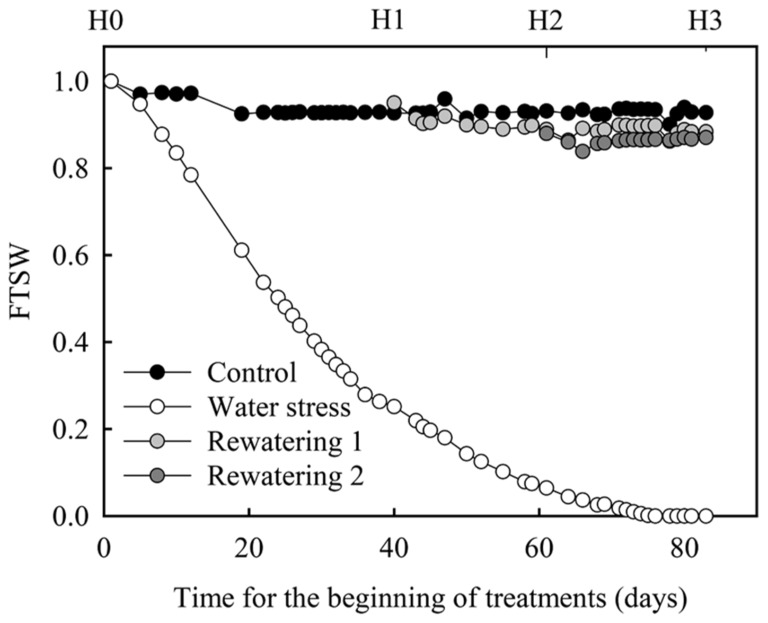
**FTSW evolution during the greenhouse experiment on oil palm.** FTSW is defined as the ratio of actual plant-available soil water (ASW) content to the total plant-available soil water (TTSW). H0, H1, H2, and H3 refer to the four harvest dates. H0 (start of the dry-down), H1 (relative transpiration of water stressed plants = 0.2 and rewatering of the plants of the rewatering 1 treatment), H2 (3 weeks after H1 and rewatering of the plants of the rewatering 2 treatment), and H3 (3 weeks after H2).

Four treatments were applied, control, water stress, rewatering 1, and rewatering 2 (**Figure 1**). For the control treatment, pots were watered using daily weighings in order to maintain soil water content at 95% of soil field capacity throughout the experiment (81 days). For the water stress treatment, no water was added to the pots throughout the experiment. For rewatering 1 treatment, no water was added to the pots until plant relative transpiration (Tr_norm) reached 0.2. When plants reached this value they were watered to maintain soil water at 95% of field capacity. For rewatering 2 treatment, no additional water was added on pots until 3 weeks after the plants reached Tr_norm = 0.2; then they were watered to maintain soil water at 95% of field capacity. FTSW was computed for each pot throughout the experiment on a daily basis (**Figure 1**). To compute FTSW, SWC_min_ was considered as the mean observed value of soil water content when Tr_norm equaled 0.1.

Along with non-destructive measurements of plant leaf area, four harvests were performed. Before the onset of the dry-down (H0) four plants were harvested. When plants reached Tr_norm = 0.2 (H1), four plants of control and water stress treatments were harvested. Three weeks after plants reached Tr_norm = 0.2 (H2), four plants of control, water stress, and rewatering 1 treatments were harvested and, 3 weeks after (H3) four plants of control, water stress, and rewatering 2 treatments were harvested. At each harvest and for each treatment, sucrose, glucose, fructose, and starch concentration were estimated on a sample of trunk, leaf lamina, and petiole. Leaf lamina and petiole samples were taken on the leaf located two ranks below the last emerging leaf. At this rank the leaf had finished its expansion and could be considered as a source for carbohydrates. During these harvests the total dry weights of leaves, petioles and trunk were also measured. Total plant NSC concentration was estimated as the ratio of (i) the sum of petioles, leaves, and trunk NSC dry weights to (ii) plant shoot dry weight. Non-structural dry weights of petioles, leaves, and trunk were computed as the product of their total dry weight and their NSC concentration. Statistical differences for NSC concentrations in the different organs between control and water stress treatments were tested by ANOVA with R (R Development Core Team, 2007).

### MODELING CONCEPTS

X-Palm (Pallas et al., 2013b) and Ecomeristem (Luquet et al., 2006) are two functional structural plant growth models respectively dedicated to palm trees and annual grasses (rice, sorghum) species. Using object-oriented modeling, both models are based on an explicit representation of plant topology and simulate plant morphogenesis as the result of meristem functioning and organ growth. At each time step of simulation (day) an index of competition (*I*_c_) is computed as the ratio of plant assimilate supply coming from photosynthesis activity to organ assimilate demand for growth and maintenance respiration. *I*_c_**modulates plant morphogenesis and topology through its effect on several developmental processes such as tillering for grasses in Ecomeristem or inflorescence sex determination or abortion in X-Palm. A reserve pool of carbohydrates is simulated by both models as a result of the supply/demand balance: in case of *I*_c_ < 1, a proportion of reserve carbohydrate biomass is mobilized to satisfy organ growth demand. Conversely, a proportion of the excess of carbohydrate is stored in the reserve pool. When *I*_c_ < 1, if reserves are missing to satisfy growth, organ growth is delayed and leaf senescence or youngest tiller abortion accelerated for annual grasses.

In both models, light interception rate is computed at the canopy level using the Beer–Lambert law and depends on a light extinction coefficient considered as genotypic. C assimilation is computed according to radiation conversion efficiency (Monteith, 1977) and the resulting C supply is partitioned among individual plants depending on cropping density. This is relevant, in this study, because both models are purely deterministic leading to an absence of variability in individual plant vegetative growth.

A water balance is daily computed (Allen et al., 1998) and enables the computation of FTSW. One of the main features of both models is that FTSW directly affects: (i) plant C assimilate supply, through the decrease in leaf transpiration and photosynthetic rates and (ii) plant C assimilate demand by a decrease in morphogenetic processes (leaf appearance and expansion rates), as previously proposed by various authors (Tardieu et al., 1999; Sinclair and Muchow, 2001; Pallas et al., 2011). The impact of FTSW on plant processes is computed using broken-stick functions as previously observed on experimental data on rice (Luquet et al., 2008; Rebolledo et al., 2013) and oil palm (Legros et al., 2009c; Pallas et al., 2013b). Genotypic differences for drought sensitivity, as observed on rice (Rebolledo et al., 2013), are taken into account using “threshold” parameters (with the suffix “_th” attached to affected process) for each process as follows:

(1) $$\frac{V\left(t\right)}{V\,\max\left(t\right)}=\{\begin{array}{cc} 1, & if\,\;FTSW(t)>\mathrm{var}iable_{-}th,\\ \frac{\begin{array}{c} FTSW\left(t\right) \end{array}}{\mathrm{var}iable_{-}th}, & else \end{array}$$

with *V*(*t*), the value of the variable (transpiration, C assimilation, expansion, leaf appearance rate) at day *t*, *V*_max_(*t*), the value of the variable in absence of water deficit and variable_th, the FTSW threshold below which the value of the variable decreases. In this study and for both models, the threshold values for leaf transpiration and C assimilation rates were identical as previously suggested for other annual plant growth models (e.g., Casadebaig et al., 2011).

#### Ecomeristem

Ecomeristem simulates plant vegetative morphogenesis of rice, sorghum, and sugarcane. The model was described in previous studies on rice plants under non-limiting (Luquet et al., 2006) and drought conditions (Luquet et al., 2012a). Regarding the rice model for vegetative phase (before internode elongation), phytomer initiation rate is scheduled by a potential plastochron (*Plasto*, genotypic parameter). It is equal to the phyllochron, i.e., the duration (in °Cd) of expansion phase of a leaf once it has appeared until the next leaf tip appears (a relationship specific to rice). Once initiated, an organ *n* is pre-dimensioned. Its potential final size is computed as the final length of leaf (*n* - 1) incremented by a genotypic parameter (meristem growth capacity, MGC). MGC quantifies the ability of the vegetative meristem to produce successive leaves with an increasing size (an allometric specific coefficient is used to translate leaf length into width; Luquet et al., 2006). This size can be down-regulated if the above mentioned variable *I*_c_**is inferior to 1. Once pre-dimensioned, a leaf expands at a leaf expansion rate (LER, cm^2^ °Cd^-^^1^) equals to the ratio between potential final leaf length and expansion duration.

Areal expansion is translated into structural dry weight demand using a leaf rank dependent value of structural specific leaf area (SLA) computed by a logarithmic equation dependent on one slope parameter, SLA*p*. As mentioned above, Ecomeristem, uses FTSW as an intermediate variable regulating plant functioning under drought (Luquet et al., 2012a): FTSW impacts directly on leaf expansion and transpiration rates and thus proportionally on C assimilation according to two broken-stick equations dependent on one threshold parameter (LER_th and Transpiration_th, for the regulation of leaf expansion and leaf transpiration rates respectively). A particularity of the model is the regulation of tillering by *I*_c_, depending on a genotypic threshold parameter *I*_ct_**(*I*_c_ threshold above which tillering occurs)*. *A genotype with low *I*_ct_ tillers more easily than one with high *I*_ct_. Root compartment is only simulated in terms of biomass, i.e., as a bulk compartment with a daily growth demand computed proportionally to that of the shoot part and depending on plant phenology.

#### X-palm

X-Palm simulates oil palm growth and yield (Pallas et al., 2013b) by accounting for the growth dynamics of each organ produced by the plant throughout its lifespan. It can simulate individual trees with different growth dynamics through stochastic rules between plant development and growth (mainly reproductive growth). This function was not used in the present study. X-Palm simulates plant vegetative and reproductive development but since the present study only addresses the drought regulation of vegetative growth, reproductive growth functions will not be detailed here. The root system biomass is not taken into account and the model only simulates root expansion in order to adjust the total transpirable soil water with plant growth. Thus in X-Palm, radiation conversion efficiency only deals with the above-ground part of the plant.

Contrary to rice, oil palm is a mono-axial plant. In X-Palm, the production of new leaves is modeled according to thermal time and depends on a genotypic parameter (*prod_rate*,**°Cd^-^^1^) representing the leaf production rate according to the daily effective temperature (daily temperature minus base temperature). Based on previous experiments the potential leaf area increase per day for each leaf is modeled using a sigmoid function describing the dynamics of leaf area expansion and using a potential final leaf area depending on genotype and plant age. Then, C assimilate demand (including growth respiration) is daily simulated for each leaf as the product of its potential leaf area increase and its chemical cost (g CH_2_O gDM^-^^1^; Pallas et al., 2013a) divided by its maximal SLA (*max_SLA*), i.e., that associated to leaf structural biomass. Internode growth is simulated in the same way considering (i) its potential volume depending on plant age and genotype, (ii) a function describing its growth dynamic, (iii) its chemical cost, and (iv) a minimal wood density (*min_dens*). As for leaf and SLA, this minimal wood density corresponds to the density associated with the structural biomass only.

At each time step of simulation if *I*_c_ > 1, potential expansion rate of each leaf and internode is achieved and a proportion (*reserve_ratio_storage*)**of the excess of biomass is allocated to the trunk (considered as a bulk reserve compartment) and leaves proportionally to their carbohydrate reserve capacity. Maximal storage capacity of trunk is modeled using one parameter (*max_NSC_content*) which accounts for the maximal concentration of NSC that can be stored in the trunk and the maximal storage capacity of leaves is modeled using a minimal SLA (*min_SLA*). The model considers the proportion of the leaf biomass included between *min_SLA* and *max_SLA* as non-structural biomass. This formalism enables computing NSC concentration in organs as the ratio of non-structural to total organ biomass.

If *I*_c_ < 1, a proportion of reserve biomass of the trunk and leaves depending on their NSC filling status is mobilized. If the amount of carbohydrate mobilized from reserves (*R*_mob_) is enough to compensate the difference between assimilate supply (*S*) and whole plant assimilate demand (*D*), internode and leaf growth is equal to their potential growth. If *R*_mob_ is not enough to compensate, all the growth shortage of day t is reported to the next days. This formalism enables to simulate the lack of plasticity observed in leaf and internode size for oil palm (Legros et al., 2009a,b). To model leaf senescence, the leaf is considered as dead when leaf thermal time from appearance exceeds a defined parameter value (active_duration**in °Cd). Previous experimental results on oil palm (Legros et al., 2009a) showed that leaf expansion was unaffected by drought whereas leaf appearance rate was strongly reduced in drought conditions. Thus, two genotypic parameters are taken into account to model these processes: (i) Leaf_appearance_th, the FTSW threshold below which leaf appearance rate decreases and (ii) Transpiration_th, the FTSW threshold below which plant transpiration decreases (and thus proportionally C assimilation). Note that the model also simulates the observed incre-ase in the leaf senescence rate in water deficit situation (Dufrêne, 1989).

The model has been already calibrated using previously published results (Dufrêne, 1989; Pallas et al., 2013b), using parameter optimization methods (Combres et al., 2013) and additional experimental data. The model was also validated under non-limiting and water deficit conditions on commonly growth commercial hybrids (Pallas et al., 2013b).

### SIMULATION EXPERIMENTS

#### Oil palm

X-Palm was run using meteorological data from La Mé (Ivory Coast 5°3′N, 3.5°′E). This site is characterized by a long dry season from December to April. Simulations were run for a period (May 1989–May 1991) with two contrasted dry seasons, a severe one (precipitation – potential evapotranspiration = -179 mm) from January to April 1990 and a low one (precipitation – potential evapotranspiration = -2 mm) from January to April 1991. To avoid bias in simulations of X-Palm caused by the stochastic relationships which generates temporal and inter-tree variations in reproductive demand (Pallas et al., 2013a), a constant reproductive demand was considered (based on the observed reproductive growth of adult oil palm; Pallas et al., 2013b) for all plants throughout the simulation using appropriate parameter values. This simplification enables analyzing the system according only to variation in vegetative demand.

#### Rice

A meteorological data set was built to consider constant daily conditions in terms of photosynthetically active radiation (7.6 MJ m^-^^2^ day^-^^1^), air temperature at plant basis (25.4 °C), and evaporative demand (1.85 mm). These values were computed as the average of those recorded during the experiment of Rebolledo et al. (2012; IRRI, Philippines, 14.1°′N, 121.2°′E). These constant daily conditions were used to avoid environmental noises and simplify the analysis of C source–sink processes for such small plants. For the simulation, plants were grown with a potential soil water reserve of 450 cm^2^ and a density of 30 plants per m^2^. One reference genotype was considered with a phyllochron of 50°Cd (i.e., among the best developmental vigor values observed in the diversity panel studied by Rebolledo et al. (2012)), moderately large leaves (MGC of 10 cm) and good tillering aptitude (*I*_ct_ of 1). Following 20 days in well-watered conditions, two drought situations were simulated: (i) a 11 day dry-down period with daily ETP of 1.85 mm (short severe stress); (ii) a 21 day dry-down (long moderate stress) with a lower ETP of 0.9 mm per day.

### SENSITIVITY ANALYSIS

For both models two parameters are directly related to the functioning of sink and source organs in response to water deficit: LER_th and Transpiration_th for rice, Leaf_appearance_th and Transpiration_th for oil palm. Accordingly, for each species and each drought condition, a sensitivity analysis in which these two parameters varied simultaneously (50 × 50 combinations) was conducted. A two way ANOVA with interaction was performed to estimate the proportion of the variance of output variables explained by both parameters and their interaction (using R software).

## RESULTS

### EXPERIMENTAL BASES

Non-structural carbohydrate plant composition showed considerable variations between the two studied species. At the onset of the dry-down, oil palm displayed a larger pool of NSC compared to rice (**Table 1**). Moreover differences were also observed between organ types and species for the relative proportion of the NSC in organs. Sucrose was the main NSC in mature leaves (source organs) for rice and oil palm (respectively 71.6 and 64.5%), whereas hexoses (50.6%) and starch (87.4%) were the main NSC respectively for young leaves in rice and trunk in oil palm (sink organs).

**Table 1:** Mean concentration of non-structural carbohydrate at the beginning of the greenhouse experiments (onset of the dry-down) for rice (genotype IR64) and oil palm (genotype G01).

		Carbohydrate concentration (mg g^-^^1^)	
Rice		**Mature leaf**	**Young leaves**
	Hexose	9.5	12.8
	Sucrose	36.7	10.5
	Starch	5.0	2.0
Oil palm		**Mature leaf**	**Trunk**
	Hexose	17.7	5.8
	Sucrose	42.3	37.2
	Starch	5.5	302.1

#### Rice

**FIGURE 2:**
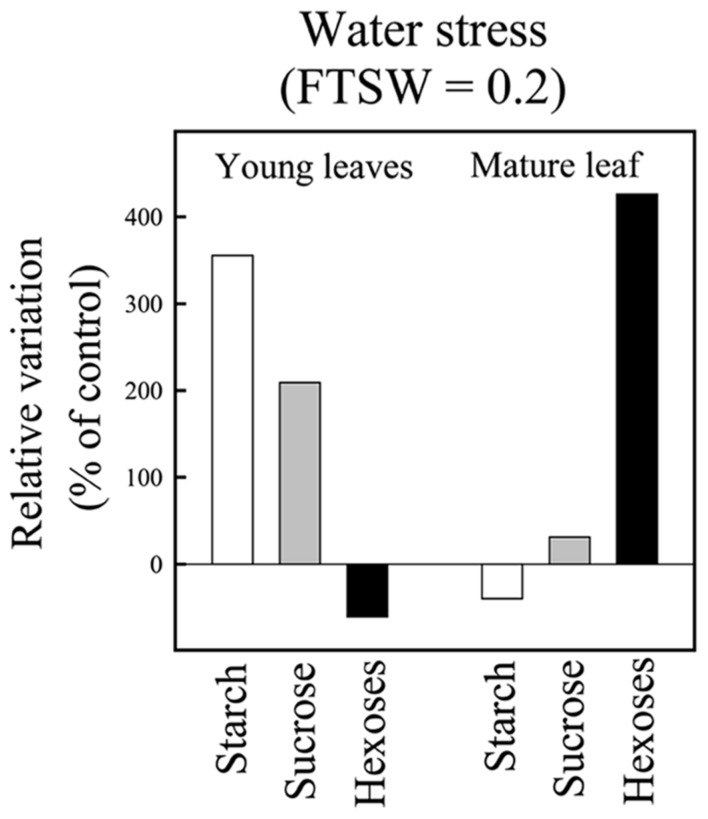
**Non-structural carbohydrate concentration in mature and young juvenile leaves at the end of the greenhouse experiment on IR64 genotype.** At the end of the experiment FTSW values reached 0.2 for the water stress plants. Each bar represents the relative variation in NSC concentrations of water stressed vs control plants. The mature leaf corresponds to the last fully expanded leaf on the main stem and the young leaves correspond to the pale green hidden expanding leaves on the main stem (adapted from Luquet et al., 2008).

Based on the detailed analysis of IR64, Luquet et al. (2008) reported that during drought establishment, organ NSC concentrations showed contrasted variations depending on leaf age (source mature vs. sink young), stress intensity, and the considered sugar. While whole plant NSC balance was not affected compared to the control treatment until a severe stress, during the dry-down, starch was progressively reduced in source (mature) leaf and increased in sink (young) leaves. It was the opposite for hexoses. Sugar concentrations are summarized in **Figure 2**, for the case of rice plants that just reached a severe stress of FTSW 0.2 (data from Luquet et al., 2008). As previously mentioned, NSC dynamics was further explored on a panel of 43 rice genotypes (Rebolledo et al., 2012) along with key variables related to plant development and expansive growth. This study confirmed the abovementioned results shown on IR64 and could relate the diversity observed on metabolic traits to that related to growth and growth maintenance under drought. In particular it was suggested that growth maintenance under drought was related to the capacity of the different genotypes to remobilize starch during the dry-down (cf. Rebolledo et al., 2012).

#### Oil palm

**FIGURE 3:**
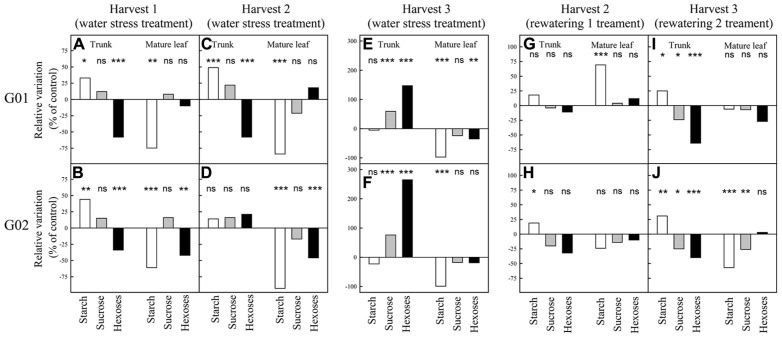
**Non-structural carbohydrate concentration in mature leaves and trunks during the experiment in greenhouse on two oil palm genotypes (G01 and G02).** Each bar represents the relative variation in NSC concentrations of water stressed (or rewatered) vs control plants. The samples for “mature leaf” correspond to the samples taken on leaf lamina of the leaf located two ranks below the last emerging leaf. See **Figure 1** for further explanations on harvest dates. Significant differences between water stress or rewatering treatments and control were tested. Significance values: *P < 0.05; **P < 0.01; ***P < 0.001; ns, not significant.

Approximately 40 days (harvest 1, FTSW ≈ 0.25, **Figures 3A,B**) and 60 days (harvest 2, FTSW ≈ 0.05, **Figures 3C,D**) after the onset of the dry-down for both genotypes starch concentration in trunk was greater in water stressed plants than in control ones whereas in mature leaves control plants displayed a significantly (*P* < 0.05) greater starch concentration (**Figures 3A–D**). By contrast, 80 days after dry-down onset (harvest 3) starch concentration in water stress plants was lower than in control plants for both types of organs and both genotypes (**Figures 3E,F**). At harvest 1 and 2, for both genotypes, sucrose concentration in leaf and trunk was not significantly different between water stress and control plants whereas this concentration was significantly greater in the trunk of water stressed plants at harvest 3. Hexose concentration in trunks was significantly lower in water stressed plants at harvest 1 (**Figures 3A,B**). At harvest 2, hexose concentration remained significantly lower in trunks in water stressed plants for G01 whereas it began to increase for G02 genotype (**Figures 3C,D**). Then, at harvest 3 (severe water stress), hexose concentration in the trunk was much higher for water stressed plants than for control ones for both genotypes (**Figures 3E,F**). At the plant scale when estimating the total NSC concentration, it was greater for water stressed than for control plants at harvest 1 and 2 for both genotypes. In contrast at harvest 3, this concentration was lower for water stress plants. For example for G01 total NSC concentration in plants was equal to 182, 191, and 119 mg g^-^^1^ respectively at harvest 1, 2, and 3 for the water stress treatment and was equal to 151, 162, and 168 mg g^-^^1^ for the control treatment. Note that the severe water stress corresponded to photosynthesis values close to 0 (data not shown).

After rewatering from moderate stress (harvest 2 for rewatering 1 treatment, **Figures 3G,H**) NSC concentrations almost returned to the control level except for starch concentration in leaves of G01 (increased compared to control) and starch concentration in trunk of G02 (increased compared to control). After rewatering from severe stress (harvest 3 for rewatering 2 treatment, **Figures 3I,J**), NSC concentration did not return to the control level in most cases. The main differences were observed for hexose concentration in trunk (decreased compared to control), for starch concentration in trunk (increased compared to control), and for starch concentration in leaves for G02 (decreased compared to control).

### SIMULATION RESULTS

#### Rice

**FIGURE 4:**
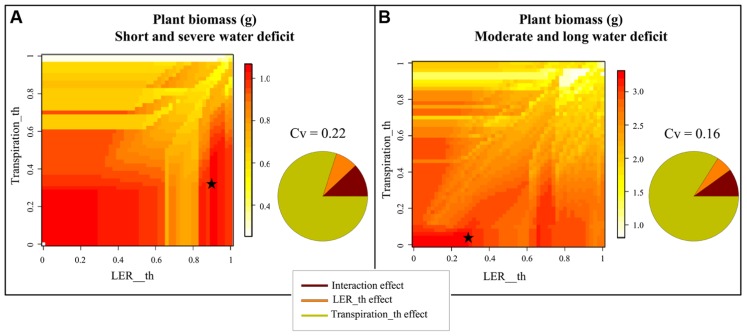
**Heatmap representation of rice seedling biomass simulated by Ecomeristem based on one genotype differing only in terms of leaf expansion and transpiration rates’ sensitivity (respectively, Transpiration_th and LER_th) to drought under (A)** strong severe and **(B)** moderate longer drought. The sensitivity analysis was carried out for 50 × 50 combinations of Transpiration_th and LER_th. Stars refer to the parameter values maximizing plant biomass for both water deficit conditions. The pie charts close to each 3D graph represent the proportion of the variance explained by both parameters and their interaction.

**Figure 4** shows the results of the sensitivity analysis performed with Ecomeristem for two contrasted drought patterns based on a simultaneous variation of parameters related to drought sensitivity of leaf growth rate (LER_th) and transpiration rate (Transpiration_th).

Under a short severe stress (**Figure 4A**), the most efficient genotypes (highest shoot green biomass) were characterized by a small Transpiration_th (between 0 and 0.4) while LER_th was either small (between 0 and 0.3) or high (between 0.85 and 0.95). The best genotype was defined by a Transpiration_th of 0.38 and a LER_th of 0.9. Under a longer moderate stress (**Figure 4B**), the most efficient genotypes were characterized by a low drought sensitivity of both LER and transpiration (Transpiration_th and LER_th inferior to 0.1 and 0.25 respectively); the best genotype was characterized by a Transpiration_th of 0 and a LER_th of 0.24. Nevertheless some efficient genotypes could present a quite high LER_th (e.g., 0.7). In both drought types, the effect of Transpiration_th was much stronger (ca. 80%) compared to that of LER_th (ca. 8%) and the interaction effect was small (ca. 10%).

**FIGURE 5:**
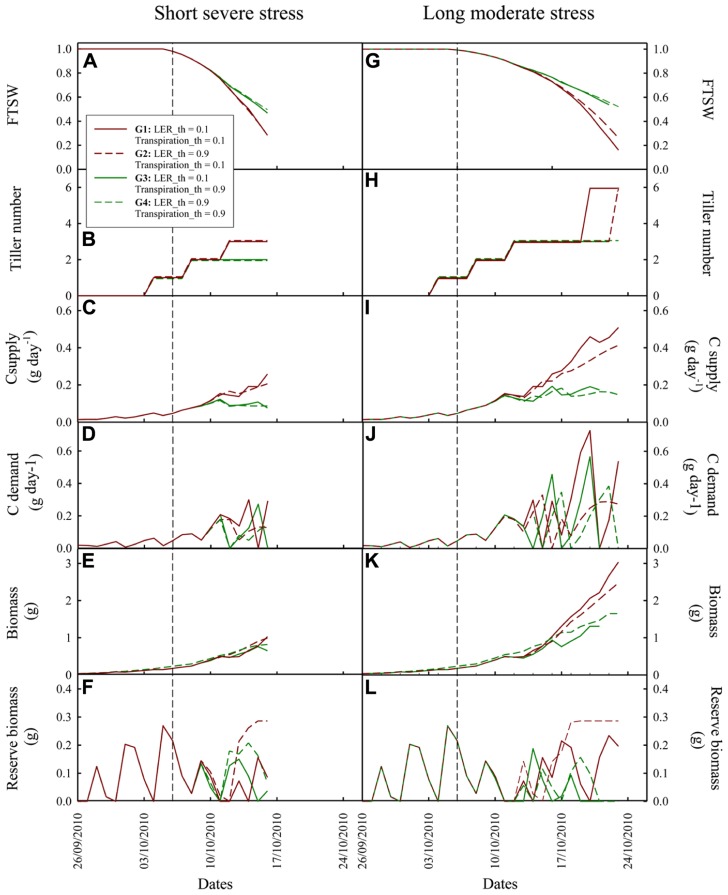
**Dynamic simulation by Ecomeristem of four rice genotypes contrasting for leaf expansion rate and leaf transpiration sensitivity to drought.** **(A–F)**: under a short severe dry-down from 20 to 31 days after germination with an evaporative demand of 1.85 mm; **(G–L)**: under a longer (20–38 days after germination) dry-down with lower evaporative demand (0.7 mm). Water stress scenarios are the same as those presented in **Figure 4**. Vertical dash lines refer to the beginning of the dry-down. See Section “Materials and Methods” for details on the simulation experiment.

**Figure 5** shows the dynamic simulation of genotypes characterized by extreme behaviors in terms of source and sink regulations. Under both drought situations the impact of LER reduction on plant biomass was generally smaller than that of transpiration reduction (cf. G3 and G4 vs. G1 and G2 in **Figures 5E–K**). The two anisohydric genotypes (G1 and G2) reached a similar level of shoot biomass accumulated at the end of the drought period. G1, with the lowest LER_th, kept on expanding leaves longer, resulting in a higher C demand (**Figures 5D,J**) and biomass supply (**Figures 5C,I**). This however generated C source–sink imbalance (cf. the lower level of C reserve for G1 compared to G2 in **Figures 5F,L**), not met by G2. Under short severe stress, this imbalance was such that it resulted in accelerated leaf senescence (cf. in **Figure 5E**, a plateau of green shoot biomass of G1 from 11 to 14/10).

The high stomatal sensitivity of G3 and G4 strongly reduced the dry-down dynamics and water use. For example, in the short severe dry-down situation (**Figure 5A**), in average, these two genotypes showed a FTSW of 0.47 at the end of the dry-down, vs. 0.28 for G1 and G2. However this soil water conservation was not sufficient to counteract the negative impact of early stomatal closure on biomass production. G3, with a low LER_th and a high Transpiration_th, kept on growing longer and finally reached severe C source–sink imbalance (cf. C demand regulation and storage; **Figures 5D,F**). This resulted in accelerated leaf senescence (cf. final reduction of green leaf biomass and area; **Figure 5E**) and plant death 3 days before the end of the dry-down (**Figure 5K**).

#### Oil palm

**FIGURE 6:**
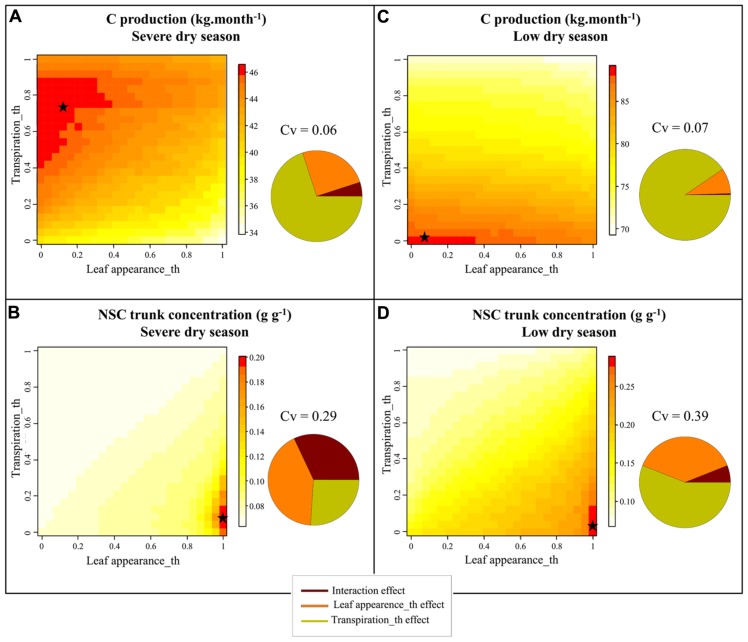
**Heatmap representation of the simulated values with X-Palm of C production (A,C)** and of non-structural carbohydrate content in trunk **(B,D)** of oil palm for 50 × 50 combinations of transpiration and leaf appearance rates sensitivity to drought (Leaf appearance_th and Transpiration_th) for the January–April 1989 [severe dry season, precipitation – potential evapotranspiration = -179 mm **(A,B)]** and January–April 1990 periods [low dry season, precipitation – potential evapotranspiration = -2 mm **(C,D)**] in La Mé (Ivory Coast). The pie charts close to each 3D graph represent the proportion of the variance explained by both parameters and their interaction. Stars refer to the parameter values maximizing plant C production and NSC contents in the trunk for both water deficit conditions.

The sensitivity analysis performed with X-Palm revealed different parameter values that maximized plant C assimilate production, for the two dry seasons differing in water deficit intensity in La Mé (severe dry seasons in January–April 1990 and low dry season in January–April 1991). For the severe dry seasons the optimal values were 0.75 and 0.15 respectively (**Figure 6A**) for the transpiration and the leaf appearance thresholds and 0.05 and 0.07 for the low dry season (**Figure 6C**). These values point out that under drought the benefit of the anisohydric and isohydric stomatal behavior depends on the intensity of soil water deficit whereas sink limitation of growth (leaf appearance rate limitation) systematically limits plant performance. Simulations also show that C assimilation per leaf area unit, directly related to plant transpiration, had a stronger impact on plant production (71 and 91% of the variance explained for the strong and low dry season respectively) than leaf appearance rate (25 and 9.1% of the variance explained). For both dry seasons, the maximal values of carbohydrate reserves were reached for leaf appearance rate threshold equal to 1 and for transpiration threshold equal to 0.10 (severe dry season) and 0.05 (low dry season; **Figures 6B,D**). The impact of the Leaf appearance_th on NSC storage was greater than on C assimilate production (42 and 35% of the variance in trunk NSC explained by this parameter under moderate and severe drought respectively).

**FIGURE 7:**
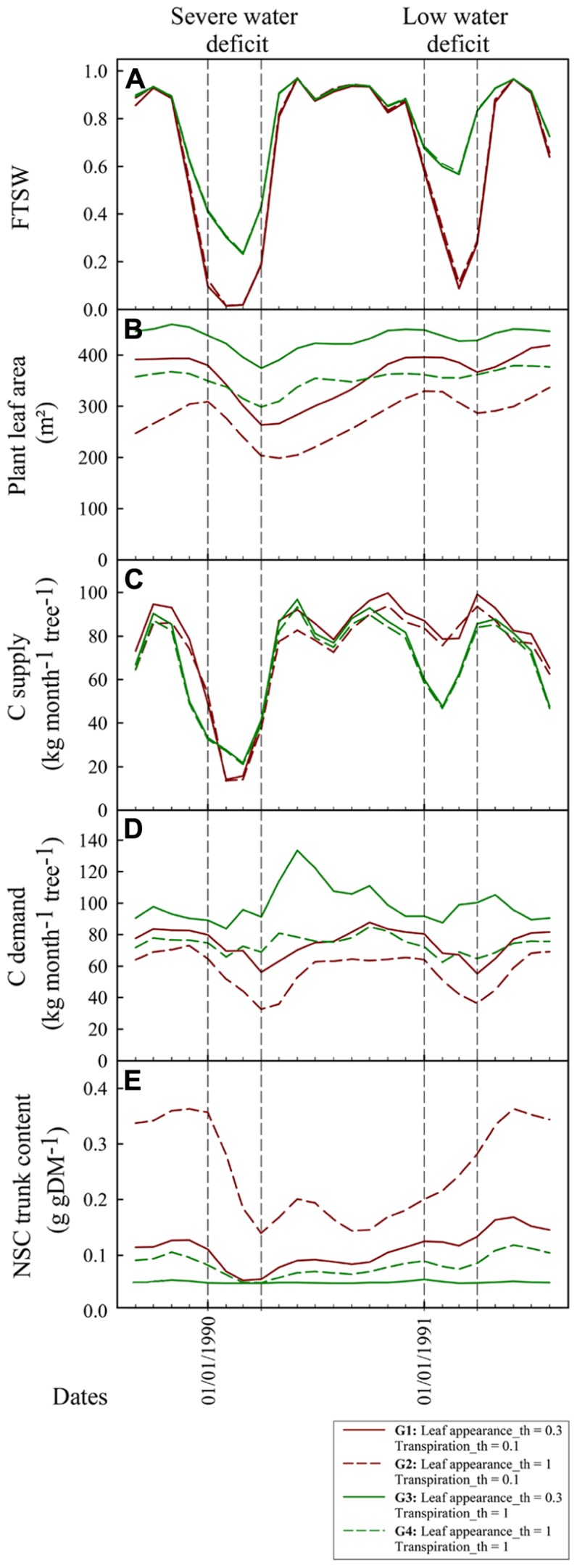
**Dynamical simulation with X-Palm of FTSW (A)**, total plant leaf area [m^2^, **(B)]**, monthly carbohydrate supply per tree [kg CH_**2**_O tree^**-**^^**1**^ month^**-**^^**1**^, **(C)**], monthly carbohydrate demand per tree [kg CH_**2**_O tree^**-**^^**1**^ month^**-**^^**1**^, **(D)**], and non-structural carbohydrate content in the trunk [g gDM^**-**^^**1**^, **(E)**] of oil palm for four genotypes contrasting for transpiration and leaf appearance rates sensitivity to drought (Leaf appearance_th and Transpiration_th). Vertical bars indicate the severe and low dry seasons presented in **Figure 6**.

**Figure 7** shows the dynamical changes in key variables describing plant functioning and growth along with the FTSW values during the May 1989–May 1991 period for four virtual genotypes (G1–G4) with different drought sensitivities for leaf appearance rate (Leaf_appearance_th) and transpiration (Transpiration_th). During the severe water deficit period, FTSW reached 0.32 and 0.02 respectively for the extreme cases of isohydric (Transpiration_th = 1, G3 and G4) and anisohydric (Transpiration_th = 0.1, G1 and G2) genotypes whereas it reached 0.58 (G3, G4) and 0.11 (G1, G2) during the low water deficit period (**Figure 7A**). The strategy to limit water use for the isohydric genotypes was not beneficial for plant production during wet seasons and the low water deficit periods (Jan – Apr 1991) whereas it was beneficial for plant production during the most severe water deficit periods (Jan – Apr 1991). C assimilate demand was smaller for the genotypes with a high leaf appearance rate sensitivity to drought (G2, G4) compared to the genotypes with a low sensitivity (G1, G3). Moreover, for G2 and C assimilate strongly decreased during water deficit periods whereas it was roughly stable for G1 and G3 (**Figure 7D**). Note that vegetative demand tended to increase after the water deficit period for the G3 genotype. This trend results from the inability of these plants to satisfy C assimilate demand for the expansion of leaves emitted during the water deficit period. As a consequence, the expansion of these leaves stopped during the water deficit period and their C assimilate demand was postponed to the wet season. The decrease in leaf area during the water deficit period resulted from two simulated processes: the decrease in leaf appearance rate, mainly observed for G2 and G4, and leaf senescence. Leaf senescence was greater for anisohydric genotypes (G1, G2) because it depends on FTSW that went down faster for these genotypes (**Figure 7A**). The amount of NSC reserves was maximal for sink limited genotypes with Leaf appeareance_th greater than Transpiration_th (G1 and G2). Nevertheless during the strong water deficit period the amount of NSC reserves also decreased for these genotypes. During this period the decrease in plant vegetative demand was not enough to offset the decrease in source activity.

## DISCUSSION

The present study illustrated how plant modeling can support experimental approaches for analyzing the interactions between water and C source–sink balances and their relative impact on plant performance. Two contrasted monocot species were addressed: oil palm, a perennial monocot characterized by a long organ lifespan and huge C reserves potentially buffering critical nutritional periods (Legros et al., 2009c); rice, an annual cereals, with relatively short organ lifespan and small C reserves with rapid turnover (Luquet et al., 2008). The same trends were confirmed for oil palm and rice. For both species, plant growth under moderate water deficit was mainly sink limited (increase of whole plant NSC concentration), whereas NSC metabolism was strongly affected in source and sinks organs. In particular it was shown that starch is preferentially stored in sink organs (**Figures 2 and 3**). Under severe stress, NSC concentration strongly decreased at the plant level and starch concentration in sink organs began to decrease. Such trends suggest that growth is also source limited under severe water deficit (**Figures 2 and 3**). To further analyze these experimental results as well as those reported by Rebolledo et al. (2012) on larger rice genetic diversity, two functional structural plant models, X-Palm (for oil palm) and Ecomeristem (for rice), both previously validated (Luquet et al., 2006, Luquet et al., 2012a,b; Pallas et al., 2013b), were applied.

### ROLE OF MODELING IN ANALYZING C AND WATER SOURCE–SINK REGULATIONS

Based on simulation experiments, this study first confirmed that an optimal plant response to drought depends on the drought type and the species considered as previously suggested by some studies (e.g., Chenu et al. (2009) reporting the environment dependent effect on maize grain yield of QTLs involved in maize LER response to drought; Heinemann et al. (2008) pointing out contrasted drought sensitivity requirements for rice depending on drought types met in Brazilian Cerrados; Pallas et al. (2011) for grapevine quantifying the optimal soil water status that leads to maximize C allocation to clusters thanks to an appropriate decrease in vegetative vigor). However, compared to previous modeling approaches (Tardieu et al., 2011), the added value of the models used here is that they formalize and help explaining, how water and C source–sink balances interact to regulate plant C use, morphogenesis, and finally performance.

For oil palm a high sensitivity of transpiration rate (**Figure 7**) was preferable under a rapid and severe soil dry-down whereas the opposite was observed under a moderate water stress. This beneficial effect of isohydric species (limiting soil water extraction) under severe water stress and of anisohydric species (maximizing soil water extraction) under moderate stress has been already reported (Tardieu and Simmoneau, 1998; Sinclair and Muchow, 2001; Andrieu et al., 2006). Meanwhile large differences in stomatal behavior were already observed for different genotypes within species (Casadebaig et al., 2008). Differences were small between the genotypes displaying a low or a high sensitivity of leaf appearance rate to drought. This relatively low impact of vegetative sink activity on plant production results probably from the fact that adult oil palms are on a relative steady state in terms of canopy development and have a large leaf area index (between 4 and 5) leading to a light interception efficiency close to 1 (Pallas et al., 2013a). Furthermore an oil palm canopy is composed of leaves produced during more than 2 years. As a consequence, decreasing leaf production during only 4 months (duration of the dry season) should not strongly modify leaf area per tree and light interception efficiency. Nevertheless, a rapid decrease in leaf appearance rate during a dry-down led to a smaller mobilization of carbohydrate reserve during water deficit or even to an increase of this reserve in situation of moderate water deficit (**Figure 7E**), as also experimentally observed on oil palm or other species (**Figure 3**; e.g., Tardieu et al., 1999). Stability in carbohydrate reserve for perennials was reported to enhance plant lifespan (Vilela et al., 2008), and this reserve pool could be mobilized to sustain the increasing carbohydrate demand for maintenance respiration when trees grow up. Since oil palm is cultivated in environments ranging from situation with a nearly absence of water deficit (South West of Asia) and situations with severe seasonal water deficits (West Africa; Corley and Tinker, 2003), the use of modeling should be a promising way for breeders to find optimal genotypic source–sink behaviors.

For rice, results are more complex as contrasted parameter combinations led to similar plant performances. In particular under a short, severe soil dry-down (**Figure 4**), the best genotypes were systematically anisohydric. Meanwhile both low and high LER_th resulted in good plant performance (**Figure 5D**). Among them, anisohydric genotypes with high LER_th (as G2 in **Figures 6A–F**) were less prone to the occurrence of C source–sink imbalance, because of earlier sink limitation, lower leaf senescence, and C reserve mobilization rates along the dry-down. This behavior should be an advantage under longer, severe drought spell, compared to genotypes maintaining leaf expansion until severe drought level, such as G1. The latter would meet, in such conditions, a situation of strong C source–sink imbalance, resulting in a lethal level of leaf senescence as previously shown on other crops (Sinclair and Muchow, 2001). Under a moderate drought, results are simpler as the virtual genotypes with the best performance are those with a low sensitivity of both transpiration and LER, which is quite similar to that observed for oil palm or other crops (Chenu et al., 2009). This type of model based analysis provides further insight to results or opinions previously reported regarding rice breeding for drought prone environments (e.g., Heinemann et al., 2008, for rice adaptation to drought types met in the Brazilian Cerrados; Courtois et al. (2000) for rainfed vs lowland cropping conditions). It provides an integrative analysis of plant processes to be combined to reach such breeding goals, which should not be apprehended experimentally because of the complexity of the system studied. With this respect, Ecomeristem can be used to explore to which extent early vigor and drought tolerance should be combined to improve rice seedling performance despite of the negative correlations pointed out between them within a rice japonica panel of 200 accessions (Rebolledo et al., 2013 using experimental data, Luquet et al., 2012a using model parameter estimation).

Ecomeristem, X-Palm and more largely appropriate functional structural plant models (FSPM) can be used for supporting the phenotypic analysis of plant growth based on its dissection into synthetic, genotypic model parameters. Such parameters can then be used as traits for genetic studies or phenotypic diversity analysis (Xu et al., 2011; Luquet et al., 2012a,b). In the same way, such models can be used to support ideotyping (optimizing model parameter combination for targeted performance and cropping environment). For these purposes, appropriate mathematical methods are needed to allow an automated generation of model parameter values and an analysis of corresponding simulation results (calibration in the case of phenotyping; criteria maximization and sensitivity analysis for ideotyping). Much progress was made in this direction recently but some improvements are still needed in order to develop methods suitable for complex system modeling (Quilot-Turion et al., 2012).

#### MODELING PROGRESSES TO ACHIEVE

The two models used in this study are particularly detailed regarding sink functioning and its genotypic variability. The formalisms used for plant C assimilate supply should be further detailed in order to deal with plant growth and C balance regulation under drought. Plant light interception efficiency and light use efficiency are formalized respectively through the Beer Lambert law (representing the plant population as a “big leaf”) and the Monteith approach (considering a crop coefficient for light conversion efficiency into biomass). These formalisms, despite their proven robustness for predicting crop performance at field (e.g., Brisson et al., 2003) are not fully relevant to deal with the elemental processes related to C assimilation that are regulated by plant nutritional status and/or environmental variables (Yin and Struik, 2009; Hammer et al., 2010; Xu et al., 2011). During the last decade many studies showed that C assimilation parameters are regulated by plant C source–sink balances in particular due to sink limitation and the subsequent accumulation of starch in photosynthesizing leaves (Paul and Foyer, 2001; Nebauer et al., 2011). In the current version of the models, the possible retroactions between sink demand and source activity are modeled in a simplistic way, as C assimilates that can neither be used for growth nor stored, are subtracted from the daily C supply (Luquet et al., 2006). This shortcoming mainly results both from missing experimental data for rice and from previous observations on oil palm showing low impact of sink activity on source functioning (Legros et al., 2009b). A coupled photosynthesis-stomatal conductance model including the impact of water stress (e.g., Egea et al., 2011) needs to be implemented in order to account for such mechanisms. First of all, further experiments are needed to quantify the genotypic variability of the elemental processes explaining photosynthetic activity differences as well as the sensitivity of these processes to abiotic constraints. Implementing a photosynthesis model should improve the simulation of the impact of water deficit, together with other abiotic constraints, on plant C assimilate supply by taking into account stomatal (diffusive) and biochemical (metabolic) limitation to photosynthesis (Egea et al., 2011). The implementation of such a coupled photosynthesis-stomatal conductance model could be a quite promising way to predict genotype production under future climates characterized by elevated temperatures and CO_2_ concentrations (Lloyd and Farquhar, 2008).

For some applications, a more detailed representation of plant and crop architecture should be also implemented. This improvement should be particularly useful (i) to model light interception at early plant stages when the Beer-Lambert law formalism is not relevant and (ii) to propose genotypic geometrical parameters (such as leaf insertion or bending angles or tillering angles) that could enhance plant potential production (Rey et al., 2008). Moreover, X-Palm and Ecomeristem are based on the principle of a whole plant C assimilate pool (Heuvelink, 1995) equally partitioned to all competitive sink organs proportionally to their demand whereas it was already reported that the physical source–sink distance can play a major role in the capacity of sinks to attract assimilate (Pallas et al., 2008; Vos et al., 2010). Because of the topological and modular representation of plants in both X-Palm and Ecomeristem, the allocation of C assimilates over the whole plant using NSC concentration gradients and assimilate transport resistances (Minchin and Lacointe, 2005) in the structure is already possible.

## CONCLUSION

In spite of abovementioned limits of the models presented in this study, it can be concluded that FSPM, if built on consistent experimental results and concepts, can help understanding and analyzing complex biological questions and the impact of genetic variability on plant performance. The importance of feedbacks between modeling and experimentations was also highlighted, particularly with respect to the challenge of exploring the impact of water and C source–sink regulations on plant agronomic vs. ecological performance. Accordingly, plant modeling should have an increasingly important role to play in support to complex trait analysis and breeding.

## Conflict of Interest Statement

The authors declare that the research was conducted in the absence of any commercial or financial relationships that could be construed as a potential conflict of interest.
